# An Intelligent Hybrid Ensemble Model for Early Detection of Breast Cancer in Multidisciplinary Healthcare Systems

**DOI:** 10.3390/diagnostics16030377

**Published:** 2026-01-23

**Authors:** Hasnain Iftikhar, Atef F. Hashem, Moiz Qureshi, Paulo Canas Rodrigues, S. O. Ali, Ronny Ivan Gonzales Medina, Javier Linkolk López-Gonzales

**Affiliations:** 1 Department of Statistics, University of Peshawar, Peshawar 25120, Pakistan; 2 Department of Statistics, Quaid-i-Azam University, Islamabad 45320, Pakistan; moiz@stat.qau.edu.pk; 3 Department of Mathematics and Statistics, College of Science, Imam Mohammad Ibn Saud Islamic University (IMSIU), Riyadh 11432, Saudi Arabia; affaragalla@imamu.edu.sa (A.F.H.); soali@imamu.edu.sa (S.O.A.); 4 Department of Statistics, University of Sindh, Jamshoro 76080, Pakistan; 5 Department of Statistics, Federal University of Bahia, Salvador 40170-110, Brazil; paulo.canas@ufba.br; 6 Facultad de Ciencias e Ingenierías Físicas y Formales, Universidad Católica de Santa María, Arequipa 04013, Peru; rgonzalesm@ucsm.edu.pe; 7 Escuela de Posgrado, Universidad Peruana Unión, Lima 15468, Peru; javierlinkolk@gmail.com

**Keywords:** breast cancer prediction, machine learning models, deep learning models, ensemble model, hybrid intelligent system, decision-making, sustainable development goals

## Abstract

**Background/Objectives:** In the modern healthcare landscape, breast cancer remains one of the most prevalent malignancies and a leading cause of mortality among women worldwide. Early and accurate prediction of breast cancer plays a pivotal role in effective diagnosis, treatment planning, and improving survival outcomes. However, due to the complexity and heterogeneity of medical data, achieving high predictive accuracy remains a significant challenge. This study proposes an intelligent hybrid system that integrates traditional machine learning (ML), deep learning (DL), and ensemble learning approaches for enhanced breast cancer prediction using the Wisconsin Breast Cancer Dataset. **Methods:** The proposed system employs a multistage framework comprising three main phases: (1) data preprocessing and balancing, which involves normalization using the min–max technique and application of the Synthetic Minority Over-sampling Technique (SMOTE) to mitigate class imbalance; (2) model development, where multiple ML algorithms, DL architectures, and a novel ensemble model are applied to the preprocessed data; and (3) model evaluation and validation, performed under three distinct training–testing scenarios to ensure robustness and generalizability. Model performance was assessed using six statistical evaluation metrics—accuracy, precision, recall, F1-score, specificity, and AUC—alongside graphical analyses and rigorous statistical tests to evaluate predictive consistency. **Results:** The findings demonstrate that the proposed ensemble model significantly outperforms individual machine learning and deep learning models in terms of predictive accuracy, stability, and reliability. A comparative analysis also reveals that the ensemble system surpasses several state-of-the-art methods reported in the literature. **Conclusions:** The proposed intelligent hybrid system offers a promising, multidisciplinary approach for improving diagnostic decision support in breast cancer prediction. By integrating advanced data preprocessing, machine learning, and deep learning paradigms within a unified ensemble framework, this study contributes to the broader goals of precision oncology and AI-driven healthcare, aligning with global efforts to enhance early cancer detection and personalized medical care.

## 1. Introduction

Breast cancer (BC) remains one of the most prevalent and life-threatening malignancies among women worldwide, characterized by the abnormal proliferation of breast cells that form malignant tumors [1,2,3,4]. If undiagnosed or untreated, these tumors can metastasize to other organs, significantly increasing mortality. In its early (in situ) stages, BC is typically non-fatal; however, in later stages, the malignant tissues originating from lobules or milk ducts invade adjacent lymph nodes and distant organs, leading to fatal outcomes. Treatment strategies for BC are patient-specific and depend on the cancer type and stage, involving combinations of surgery, chemotherapy, radiotherapy, and hormonal therapy. Although BC affects both sexes, women are disproportionately impacted, with men accounting for only 0.5–1% of total cases and typically receiving similar treatment regimens. Several risk factors, including age, alcohol consumption, obesity, radiation exposure, genetic predisposition, and hormonal therapy, increase the likelihood of developing BC [5,6,7,8,9].

Globally, approximately 2.3 million new BC cases were reported among women in 2020, resulting in about 685,000 deaths. In the preceding five years, nearly 7.8 million women were diagnosed, making BC the most prevalent cancer worldwide. The mortality trend has shifted over the decades: while it increased steadily from the 1930s to the 1970s—when surgery was the only treatment option—it began to decline in the 1990s due to the establishment of early detection programs, improved screening, and the introduction of effective multimodal therapies [10,11,12,13]. Between the 1980s and 2020, mortality from age-standardized BC dropped by 40% in developing countries, while high-income nations achieved an annual reduction of 2–4% through strengthened healthcare systems, structured referral networks, and comprehensive treatment protocols. These improvements underscore the importance of early detection, efficient referral systems, and access to specialized oncology care. The World Health Organization’s (WHO) Global Breast Cancer Initiative (GBCI) aims to reduce BC mortality by 2.5% annually, potentially averting 2.5 million deaths globally between 2020 and 2040 [10,14,15,16]. Achieving these objectives requires not only early diagnosis and timely treatment but also the integration of advanced, data-driven diagnostic systems into healthcare infrastructures.

In recent years, statistical and machine learning (ML) algorithms have been increasingly applied for disease prediction, classification, and prognosis across various medical domains [15,17,18,19,20]. For BC, ML, and deep learning (DL) models have demonstrated notable success in predicting disease progression, identifying genetic markers, and supporting diagnostic decisions. Traditional methods such as Cox proportional hazards regression, Support Vector Machines (SVMs), and random survival forests (RSFs) have been widely used for survival analysis and outcome prediction. For example, Dehdar et al. [21] compared four prognostic models—SVM, RSF, Cox regression, and Cox elastic net (Cox-EN)—for predicting survival in BC patients at Fudan University’s Shanghai Cancer Center, reporting the superior performance of RSF. Similarly, Thakur et al. [22] applied five ML algorithms, including artificial neural networks (ANNs), Random Forests (RFs), decision trees (DTs), AdaBoost, and SVM, to the METABRIC dataset, revealing that ANN was the most accurate (87.43%) for BC classification. Other studies have identified genetic markers for triple-negative BC using DT, RF, and set covering machines [23,24]. Furthermore, numerous comparative studies [25,26,27,28] have evaluated algorithms such as K-nearest neighbors (KNN), naïve Bayes (NB), Logistic Regression (LGR), multilayer perceptron (MLP), and SVM using the Wisconsin Breast Cancer Dataset, consistently demonstrating the potential of ML-based diagnostic systems to improve predictive reliability.

Despite these advances, a research gap persists in developing intelligent ensemble learning frameworks for breast cancer prediction. Ensemble learning enhances model robustness and predictive power by integrating multiple weak and strong learners to form a unified decision system. To address this gap, the present study proposes an intelligent hybrid system designed to improve the accuracy and efficiency of BC prediction. The proposed system combines a data normalization technique (min–max), a data balancing strategy (Synthetic Minority Over-sampling Technique, SMOTE), and a suite of ML, DL, and novel ensemble models. The proposed framework follows three main stages: (1) preprocessing and balancing raw datasets, (2) applying ML, DL, and ensemble models to filtered datasets, and (3) assessing model consistency under three distinct training–testing scenarios.

The models are evaluated using six performance indicators, graphical diagnostics, and statistical significance testing. Comparative analysis reveals that the proposed ensemble model significantly outperforms standalone ML and DL models and achieves results superior to those of the best-performing models in the existing literature. To the best of our knowledge, no prior study has introduced such a comprehensive hybrid system for BC prediction.

The remainder of this article is organized as follows: Section 2 describes the proposed intelligent ensemble learning framework; Section 3 presents the experimental results; Section 4 provides a comparative analysis with leading models in the literature; and Section 5 concludes with key findings, limitations, and future research directions.

## 2. Methods and Materials

This section presents the methodological framework, data preprocessing techniques, and models used to develop the proposed intelligent ensemble framework for breast cancer (BC) prediction. The framework integrates both machine learning (ML) and deep learning (DL) models, optimizing them through systematic data normalization and class rebalancing to enhance diagnostic accuracy and reliability. The design of this framework aligns with the goals of multidisciplinary cancer healthcare, combining computational intelligence with medical diagnostic insights to improve decision support in breast cancer detection.

### 2.1. Data Description and Preprocessing

The experimental data used in this study were obtained from the publicly available UCI Machine Learning Repository [29]. The version utilized in this study simply includes the pre-extracted numerical characteristics available in Excel format, despite the fact that the original WDBC dataset was produced from digitized fine-needle aspirates (FNAs) of breast tissue, which comprises 569 instances categorized into two diagnostic classes: benign and malignant tumors. Each sample is described by 32 attributes (see Table A1), including an identification number, a diagnosis label, and 30 real-valued features representing tumor morphology (e.g., radius, texture, perimeter, area, smoothness, compactness, concavity, concave points, symmetry, and fractal dimension). Among these, 357 samples are benign and 212 are malignant, indicating a moderate class imbalance. More detailed information about this dataset can be found in [30].

Before model training, data preprocessing is a crucial step to ensure that the predictive models operate efficiently and without bias. The preprocessing pipeline implemented in this work consists of two major components: (1) feature scaling and normalization, and (2) data balancing to address class imbalance.

#### 2.1.1. Data Normalization

The first step is to scale all input variables to a common range to prevent features with larger magnitudes from dominating the learning process. In this study, the min–max normalization technique was employed, which linearly scales each feature into the range [0,1]. The normalization function is expressed as

(1) $$f\left(y\right)=\frac{y-\min(y)}{\max(y)-\min(y)}$$

where max(y) and min(y) represent the maximum and minimum values of the feature vector *y*, respectively. This transformation enhances model convergence and stability, particularly for distance-based algorithms.

#### 2.1.2. Handling Class Imbalance

Class imbalance can severely bias predictive models toward the majority class, leading to suboptimal detection of malignant cases—an outcome with significant clinical implications [31]. In the present study, the dataset exhibited an imbalance ratio of approximately 1.68:1 between benign and malignant samples. To mitigate this issue, a hybrid resampling approach combining K-Means-based Synthetic Minority Over-sampling Technique (K-Means SMOTE) and random under-sampling was employed.

The K-Means SMOTE algorithm enhances the traditional SMOTE by introducing a clustering phase that partitions the minority class into homogeneous subclusters before generating synthetic samples. This ensures that over-sampling occurs within well-defined feature spaces, reducing the risk of noise amplification and improving the representation of class boundaries. The following pseudo-code Algorithm 1 summarizes the procedure.
**Algorithm 1** K-Means SMOTE for minority class over-sampling.**Require:** Minority class dataset $D_{m}=\left\{x_{1},x_{2},\ldots ,x_{n}\right\}$, number of clusters $k\in Z^{+}$, over-sampling factor *N***Ensure:** Synthetic dataset $D_{\mathrm{syn}}$ to augment $D_{m}$
1:**Clustering Phase:**Apply K-Means clustering to $D_{m}$ to partition it into *k* disjoint clusters: $C=\{C_{1},C_{2},\ldots ,C_{k}\}$.2:**Sample Allocation Phase:**For each cluster $C_{i}$, compute the number of synthetic samples $n_{i}$ to generate, proportionally to cluster size $|C_{i}|$.3:**Synthetic Sample Generation:**4:**for** each cluster $C_{i}\in C$ **do**5:    Compute the centroid $\mu_{i}=\frac{1}{|C_{i}|}\sum_{x\in C_{i}}x$.6:    **for** each instance $x\in C_{i}$ **do**7:        Find the set of *k* nearest neighbors $N_{k}\left(x\right)$ within $C_{i}$.8:        **for** j=1 to $\left\lfloor n_{i}/|C_{i}|\right\rfloor$ **do**9:           Randomly sample $x_{nn}\in N_{k}\left(x\right)$.10:           Generate a synthetic sample: $x_{\mathrm{syn}}=x+\lambda \left(x_{nn}-x\right)$, where λ∼U(0,1).11:           Add $x_{\mathrm{syn}}$ to $D_{\mathrm{syn}}$.12:        **end for**13:    **end for**14:**end for**15:**return** Augmented dataset $D^{\prime }=D_{m}\cup D_{\mathrm{syn}}$.

This hybrid balancing strategy ensures equitable representation of both tumor classes, thereby preventing diagnostic bias and improving the clinical applicability of the trained models. Such preprocessing steps are critical for ensuring fairness, transparency, and reproducibility in AI-assisted healthcare systems.

### 2.2. Predictive Modeling Framework

After the dataset is normalized and balanced, the prepared data are used to train and evaluate three traditional ML models, three DL architectures, and a proposed ensemble model that combines their predictive strengths. Specifically, the implemented models include the following:

- Machine Learning Models: Logistic Regression (LGR), Support Vector Machine (SVM), and Random Forest (RF);
- Deep Learning Models: AlexNet, Gated Recurrent Unit (GRU), and Bidirectional Gated Recurrent Unit (BiGRU);
- Proposed Ensemble Model: a hybrid integration of ML and DL models designed to exploit complementary learning representations.

Each model is trained independently on the preprocessed dataset, and hyperparameters are optimized via cross-validation. The proposed ensemble approach integrates probabilistic and feature-level fusion to enhance predictive robustness and clinical interpretability. The ensemble’s design aims to reduce individual model variance, improve sensitivity to malignant cases, and yield a more generalizable diagnostic framework suitable for deployment in clinical decision-support systems.

This multidisciplinary design bridges computational modeling, data engineering, and clinical oncology, representing a step toward intelligent, data-driven cancer diagnostics in modern healthcare ecosystems.

### 2.3. Predictive Models

To enhance diagnostic precision and clinical decision-making in breast cancer (BC) care, this study integrates a multidisciplinary ensemble framework combining classical machine learning (ML) and deep learning (DL) models. The framework aims to leverage complementary strengths across statistical, tree-based, and neural approaches to achieve robust, interpretable cancer prediction. Three ML models (Logistic Regression, Support Vector Machine, and Random Forest), three DL models (AlexNet, Gated Recurrent Unit (GRU), and Bidirectional GRU (BiGRU)), and their proposed ensemble model were developed and evaluated on the processed Wisconsin Breast Cancer dataset.

#### 2.3.1. Machine Learning Models

##### Logistic Regression (LR)

LR serves as a baseline probabilistic classifier that models the likelihood of malignancy as a logistic function of input predictors. The model computes the posterior probability $P\left(y=1|X\right)=\frac{1}{1+e^{-(\alpha_{0}+\sum_{i=1}^{p}\alpha_{i}X_{i})}}$, where $\alpha_{i}$ denotes feature coefficients. Despite its simplicity, LR offers interpretability and is widely used in clinical prediction due to its capacity to estimate odds ratios for risk factors [32].

##### Support Vector Machine (SVM)

SVM identifies an optimal separating hyperplane that maximizes the margin between benign and malignant cases. Using a linear kernel $K\left(x_{i},x_{j}\right)=x_{i}^{⊤}x_{j}$, the model effectively handles high-dimensional data while mitigating overfitting [20]. This makes it suitable for structured biomedical data with distinct class boundaries.

##### Random Forest (RF)

RF is an ensemble of decision trees trained on bootstrap samples with random feature selection. The final classification is obtained via majority voting across trees:

$${\hat{y}}_{RF}\left(X_{\mathrm{new}}\right)=\mathrm{mode}\left\{T_{1}\left(X_{\mathrm{new}}\right),T_{2}\left(X_{\mathrm{new}}\right),\ldots ,T_{n}\left(X_{\mathrm{new}}\right)\right\}.$$

RF is resilient to noise and provides feature importance insights that enhance clinical interpretability.

#### 2.3.2. Deep Learning Models

##### AlexNet

AlexNet is a convolutional neural network (CNN) composed of five convolutional and three fully connected layers, utilizing ReLU activation and dropout regularization. The CNN can identify feature interactions through its convolutional layers by using the AlexNet algorithm to transform the n-dimensional feature vector into a two-dimensional image-like representation (such as a 6×6 matrix or encoded image). It efficiently extracts spatial hierarchies from high-dimensional biomedical data such as cytology or histopathology images [33]. The final classification employs a softmax layer to estimate malignancy probabilities.

##### Gated Recurrent Unit (GRU)

GRU, a type of recurrent neural network, mitigates the vanishing gradient problem and captures temporal dependencies in sequential data. It employs update ($z_{t}$) and reset ($r_{t}$) gates to regulate information flow:

$$h_{t}=\left(1-z_{t}\right)⊙h_{t-1}+z_{t}⊙{\tilde{h}}_{t}.$$

GRUs are particularly effective for patient time-series data, capturing evolving diagnostic indicators [34].

##### Bidirectional GRU (BiGRU)

BiGRU extends GRU by processing input sequences in both forward and backward directions, yielding richer contextual understanding:

$$h_{t}=[{\vec{h}}_{t}\;||\;{\overset{\leftarrow }{h}}_{t}].$$

This bidirectional learning structure improves prediction stability and is well-suited for medical datasets where both prior and subsequent observations influence diagnostic inference [35].

#### 2.3.3. Proposed Ensemble Model

Ensemble learning is a powerful paradigm in computational oncology, enabling the integration of heterogeneous predictive models to achieve higher diagnostic precision and robustness [36,37,38]. Within the context of breast cancer (BC) prediction, where diverse biological, clinical, and imaging features interact nonlinearly, single-model frameworks often face limitations in capturing the full complexity of disease dynamics. Hence, a multidisciplinary ensemble approach is adopted to combine both machine learning (ML) and deep learning (DL) paradigms—leveraging their complementary strengths to improve diagnostic accuracy, generalizability, and clinical trustworthiness.

The proposed ensemble integrates six distinct base learners: three ML models—Logistic Regression (LGR), Support Vector Machine (SVML), and Random Forest (RF)—and three DL models—AlexNet, Gated Recurrent Unit (GRU), and Bidirectional GRU (BiGRU). The ML models contribute interpretability and feature-level transparency, whereas the DL models capture nonlinear hierarchical relationships and temporal dependencies within the diagnostic data. This hybrid synergy aligns with the objectives of multidisciplinary cancer analytics, bridging algorithmic rigor and clinical relevance.

A validation-based performance weighting scheme is employed to aggregate the model outputs into a unified diagnostic decision. Let ${\hat{y}}_{i}$ denote the prediction of the $i^{th}$ base model (i=1,2,…,6), and $w_{i}$ its associated confidence weight determined through validation performance metrics such as F1-score or AUC. The ensemble prediction ${\hat{y}}_{\mathrm{ensemble}}$ is expressed as a convex combination of the individual model predictions:

(2) $${\hat{y}}_{\mathrm{ensemble}}=0.1511\cdot {\hat{y}}_{1}+0.2151\cdot {\hat{y}}_{2}+0.1179\cdot {\hat{y}}_{3}+0.1912\cdot {\hat{y}}_{4}+0.1329\cdot {\hat{y}}_{5}+0.1818\cdot {\hat{y}}_{6}$$

subject to the normalization constraint:

$$\sum_{i=1}^{6}w_{i}=1,\;w_{i}>0\;\forall i.$$

Each $w_{i}$ reflects the relative diagnostic reliability or predictive contribution of model *i*, calibrated through cross-validation (k-fold cross-validation (k = 10) with Adam optimizer with a learning rate of 0.001) performance on held-out subsets of the dataset. Higher weights correspond to models demonstrating superior stability, sensitivity, or specificity in BC classification.

This weighted averaging mechanism allows the ensemble to balance interpretability and deep representational capacity—enhancing both model fidelity and clinical utility. By uniting statistical learning and neural inference under a unified ensemble framework, the model supports oncological decision-making with improved robustness against overfitting and reduced diagnostic uncertainty. Furthermore, such an integrative approach underscores the essence of multidisciplinary cancer care, in which computational, clinical, and diagnostic insights converge to enable more personalized, evidence-based cancer management.

### 2.4. Performance Metrics

To ensure the robustness and clinical reliability of the proposed intelligent hybrid system for breast cancer prediction, model performance was quantitatively assessed using six key evaluation metrics: accuracy, sensitivity, specificity, F1-score, Brier score, and error rate. Additionally, the Diebold–Mariano (DM) test was employed to assess the predictive equivalence of competing models, while visual diagnostics (bar, line, and level plots) were used for comparative analysis.

The performance indicators are defined as follows:

(3) $$\begin{array}{cccccc} \mathrm{Accuracy} & =\frac{\mathrm{TP}+\mathrm{TN}}{\mathrm{TP}+\mathrm{FP}+\mathrm{TN}+\mathrm{FN}}, & \mathrm{Sensitivity} & =\frac{\mathrm{TP}}{\mathrm{TP}+\mathrm{FN}}, & \mathrm{Specificity} & =\frac{\mathrm{TN}}{\mathrm{TN}+\mathrm{FP}}, \end{array}$$

(4) $$\begin{array}{cccccc} F1\text{-}\mathrm{Score} & =2\times \frac{\mathrm{Precision}\times \mathrm{Recall}}{\mathrm{Precision}\;+\;\mathrm{Recall}}, & \mathrm{Brier}\;\mathrm{Score} & =\frac{1}{n}\sum_{i=1}^{n}{\left(y_{i}-{\hat{y}}_{i}\right)}^{2}, & \mathrm{Error} & =(y_{i}-{\hat{y}}_{i}), \end{array}$$

where TP, TN, FP, and FN denote true positive, true negative, false positive, and false negative values, respectively. Lower Brier scores and error rates indicate better predictive reliability, while higher values of accuracy, sensitivity, specificity, and F1-score indicate enhanced model effectiveness in clinical classification tasks.

(5) $${\mathrm{DM}}_{s}=\frac{\bar{y}}{\sqrt{\mathrm{Var}(\bar{y})}},$$

where $\bar{y}$ is the mean loss differential and $\mathrm{Var}(\bar{y})$ denotes its variance. A significant DM statistic indicates that the predictive performance of two competing models differs meaningfully.

However, these metrics and statistical evaluations provide a multidisciplinary framework for assessing both the algorithmic efficiency and the clinical reliability of breast cancer prediction models, ensuring that the proposed hybrid ensemble system meets rigorous healthcare standards for diagnostic decision support.

**Figure 1:**
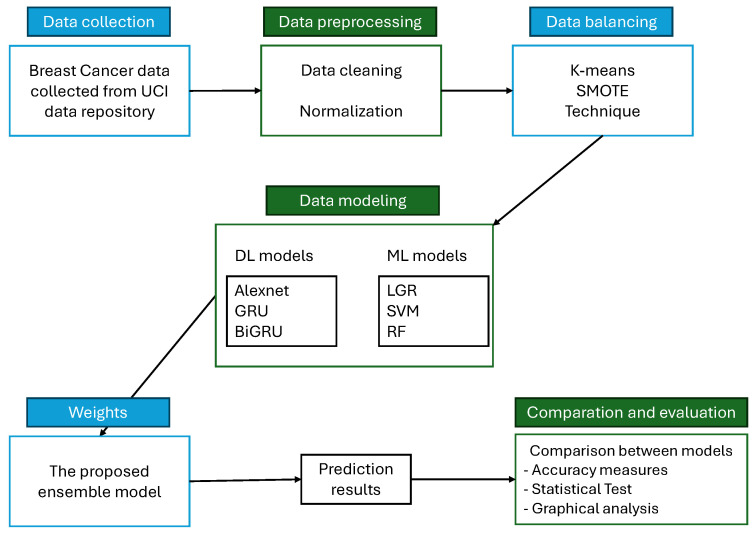
A flowchart illustrates a systematic pipeline for predicting breast cancer.

Finally, this study verifies the superiority of the proposed ensemble learning model using bar and level plots. Therefore, to conclude this section, the algorithm for the proposed hybrid intelligent prediction system, shown in Algorithm 2, is presented in Figure 1.
**Algorithm 2** Hybrid intelligent prediction system for breast cancer classification.**Require:** Raw breast cancer dataset D from the USI Machine Learning Repository [29]**Ensure:** Optimized ensemble classification performance and evaluation metrics1:**Preprocessing Phase:****Class Balancing:** Apply SMOTE-KMeans over-sampling (see Algorithm 1) to address class imbalance in D, generating a balanced dataset $D^{\prime }$.**Normalization:** Scale numerical features in $D^{\prime }$ using min–max normalization, yielding the transformed dataset $D^{\prime \prime }$.2:**Model Training Phase:****Supervised Learning Models:** Train the following classifiers on $D^{\prime \prime }$:
−(LR)−Linear kernel (SVM)−(RF)**Deep Learning Models:** Initialize and train the following neural architectures:
−Alexnet(GRU)(BiGRU)**Ensemble Model:** Construct a weighted ensemble model E combining all six base learners. Assign weights $w=[w_{1},w_{2},\ldots ,w_{6}]$ proportional to each model’s performance is presented in Figure 2.3:**Experimental Setup:**Split the dataset into three train/test configurations:
−$S_{1}$: 90% training, 10% testing−$S_{2}$: 75% training, 25% testing−$S_{3}$: 50% training, 50% testingFor each split $S_{i}$, repeat model training and evaluation for R=500 independent simulation runs to ensure statistical reliability and robustness.4:**Performance Evaluation:**Assess all models using:
−Six standard performance metrics−Graphical performance visualization (e.g., bar plot, line plot, and level plot)−Statistical significance testing (e.g., the Diabold-Marino test) to validate comparative model performance

**Figure 2:**
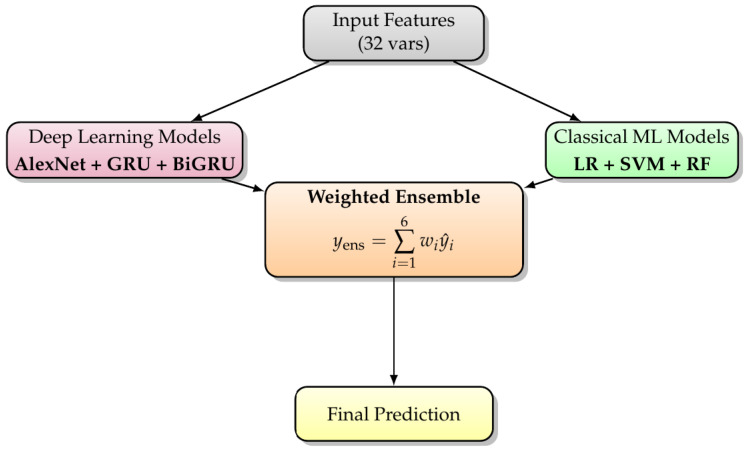
Compact flow diagram of the weighted ensemble classification model.

### 2.5. Computational Environment

This section offers comprehensive details about the computational environment, the libraries utilized, and the parameters implemented throughout the research. By providing this technical information, we aim to enable fellow researchers to replicate our results and confidently build upon this work with fidelity. The subsequent Table 1 and Table 2 detail the precise computational tools and setups used in our analysis.

## 3. Prediction Results of the Intelligent Hybrid System

Section 3 presents and discusses each model’s performance in detail. The results are thoroughly clarified in this section, along with how they were assessed using error metrics. The primary objective of this research is to predict BC patients with the developed intelligent hybrid system. To do this, the dataset used in the current research study is sourced from the USI machine learning repository [29]. On the other hand, when working on the primary dataset modeling, it is necessary to prepare the data to simplify the modeling and prediction process. Therefore, in the proposed intelligent hybrid prediction system, the following steps are followed: (1) to prepare the raw dataset by normalizing and balancing it; (2) to use the filtered dataset to implement three machine learning, deep learning, and their proposed ensemble model; and (3) to verify the consistency of the considered predictive models, splitting the data into three training and testing scenarios—(90%, 10%), (75%, 25%), and (50%, 50%)—with the simulation being run five hundred times to verify the consistency and generaliziblity of the models, which are comparatively evaluated by six various performance measures (accuracy, sensitivity, specificity, F1-score, Brier score, and error rate), a graphic analysis (a bar plot, a line plot, and a level plot), and equal prediction statistical test (a Diebold–Marino test). The details about these evaluation criteria for each training and testing scenario are as follows.

However, Table 3 presents a comprehensive comparison of the performance of various predictive models under three distinct data distribution scenarios: 50%–50% (balanced training and testing), 75%–25% (moderately imbalanced training and testing), and 90%–10% (highly imbalanced training and testing). The analyzed models include SVM, RF, LGR, AlexNet, GRU, BiGRU, and an ensemble model, with performance measured using accuracy, sensitivity, specificity, F1-score, Brier score, and error rate.

To ensure that the accuracy of the model does not change due to the data percentage, balanced and unbalanced training and testing partitions were compared. As a consequence, evaluating both configurations helps confirm that generalization and model reliability are independent of a particular split configuration.

In the first scenario (50%–50%)—Balanced Data (training and testing)—the ensemble model achieves the highest accuracy (0.9894), surpassing all other models. It also demonstrates the highest sensitivity (0.9715) and specificity (0.9814), showing its efficiency in accurately identifying both positive and negative cases. Additionally, the highest F1-score (0.9774) among all models reflects a favorable precision–recall balance. The Brier score (0.0187) and error rate (0.0106) are also the lowest, underscoring the model’s strong reliability. In contrast, among individual models, AlexNet (0.9739 accuracy) is the best performer, followed by GRU (0.9603) and BiGRU (0.9644), which exhibit robust deep learning capabilities. However, traditional machine learning models, such as LGR (0.9504 accuracy) and RF (0.9476 accuracy), fall short, suggesting that deep learning models are better suited for this predictive task.

In the second scenario (75%–25%)—Moderately Imbalanced Data (training and testing)—as the data distribution shifts towards imbalance, the ensemble model retains its top position with the highest accuracy (0.995), alongside the best sensitivity (0.9771), specificity (0.987), and F1-score (0.983). The lowest error rate (0.005) and Brier score (0.0161) further emphasize its strength in handling data imbalance. Meanwhile, AlexNet (0.9795 accuracy) and GRU (0.9659 accuracy) continue to perform well, while BiGRU (0.97 accuracy) also shows solid predictive capability. Traditional models, such as RF (0.9532 accuracy) and LGR (0.956 accuracy), encounter more challenges in this scenario due to their heightened sensitivity to class imbalance.

In the third scenario (90%–10%)—Highly Imbalanced Data (training and testing)—in the most severe imbalance condition, the ensemble model remains highly effective with the highest accuracy (0.9976), sensitivity (0.9814), and specificity (0.9913). It also achieves the best F1-score (0.987), the lowest Brier score (0.0141), and the lowest error rate (0.0024), demonstrating its ability to generalize well, even when class distributions are extremely skewed. Among individual models, AlexNet (0.9821 accuracy) and GRU (0.9627 accuracy) still perform admirably, while RF (0.9558 accuracy) and LGR (0.9586 accuracy) experience a decline in predictive reliability due to the severe imbalance.

In all three scenarios, the ensemble model consistently surpasses each model concerning accuracy, sensitivity, specificity, and F1-score while achieving the lowest error rate and Brier score. This demonstrates that combining multiple models yields more reliable predictions, particularly when dealing with imbalanced datasets. Conversely, among the individual models, deep learning architectures (AlexNet, GRU, and BiGRU) exhibit superior performance compared to traditional machine learning models (SVM, RF, and LGR). GRU and BiGRU tend to show slightly better sensitivity but have higher error rates than AlexNet. RF and LGR face greater challenges as data imbalance intensifies, underscoring their shortcomings in handling skewed distributions. The ensemble model proves to be the most dependable in all scenarios, achieving the highest overall performance. Deep learning models, especially AlexNet, GRU, and BiGRU, consistently outperform traditional machine learning models. Traditional ML models (SVM, RF, LGR) exhibit lower accuracy and struggle with class imbalance, rendering them less effective for imbalanced datasets. Addressing class imbalance is essential, as evidenced by the deterioration in performance of specific models when transitioning from a balanced to an imbalanced dataset. The ensemble strategy is a successful method to enhance predictive performance while ensuring robustness across varying data distributions. This analysis highlights the significance of adopting an ensemble technique for complex predictive tasks, as it capitalizes on the strengths of multiple models to achieve exceptional results in both balanced and imbalanced data scenarios.

On the other hand, after evaluating the model’s performance using accuracy metrics (accuracy, sensitivity, specificity, F1-score, BS, and error), the same prediction statistical tests (DM tests) were employed to assess the significance of differences in predictive ability between models. Table 4 shows the results of the DM test of each model pair to quantify the outcome quality (mean performance measurement). The results of the DM test (*p*-values) are shown in Table 4. The table confirms that the ensemble model is statistically significant at the 5% level compared to the other six prediction models.

Furthermore, this study employed the Wilcoxon signed-rank test in Table 5 to confirm that the ensemble model outperformed the individual machine learning and deep learning models across all evaluation scenarios (*p* < 0.05). These results validate that ensemble integration improves the model’s generalization and stability, thereby bolstering its application for accurate and dependable breast cancer prediction.

**Figure 3:**
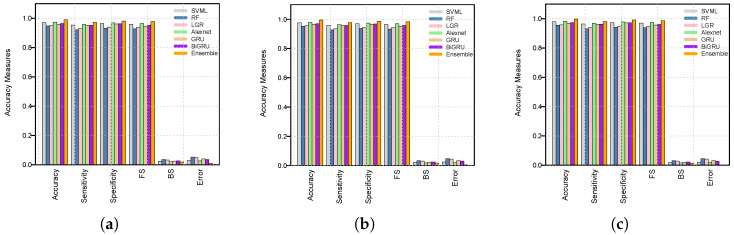
Performance measure bar plots for all predictive models for all three scenarios: (**a**) 50% training and 50% testing, (**b**) 75% training and 25% testing, (**c**) 90% training and 10% testing.

**Figure 4:**
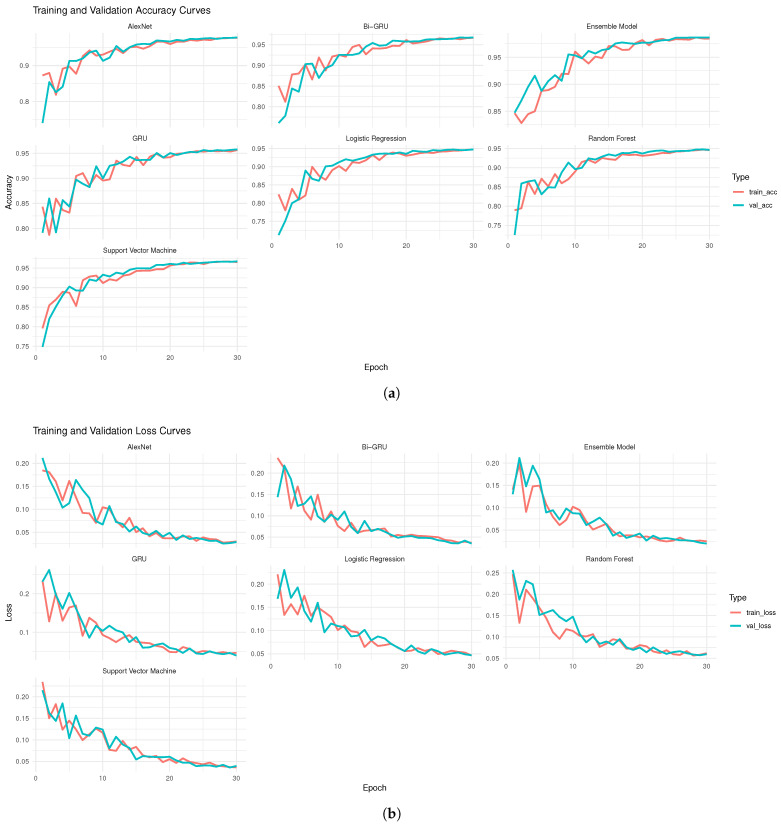
Curves for training and validation accuracy and loss: (**a**) training and validation curves; (**b**) error loss curves.

In addition to the accuracy measures analysis, the models’ performance was explored graphically in bar plots shown in Figure 3 for all three training and testing scenarios: (90%, 10%), (75%, 25%), and (50%, 50%). For instance, Figure 3a displays performance matrices of accuracy, sensitivity, specificity, F-1 score, BS, and error of first scenario 50% training and 50% testing for the used models. In this figure, the gray bar represents SVML, light black for RF, pink for LGR, light green for Alexnet, light pink for GRU, purple for BiGRU, and orange for ensemble, respectively. Thus, the bar plot confirms that the ensemble model outperformed the others, followed by the second-best AlexNet model. However, the second and third scenarios are displayed in Figure 3b—75% training and 25% testing set—and in Figure 3c—90% training and 10% testing set—respectively. These figures also illustrate that the ensemble model achieved the best performance among all models. However, these figures also confirmed that the RF and LGR models performed worst in overall BC tissue malignancy prediction scenarios. Thus, the superiority of the ensemble is also confirmed by graphical analysis. This study also presents the accuracy and loss curves of the ML and DL models along with the proposed ensemble model in Figure 4. All models’ accuracy and loss curves in Figure 4 show consistent and reliable learning behavior. The training and validation accuracies of models like LR, SVM, and RF increase smoothly and converge closely. At the same time, the associated loss curves slowly decrease, indicating optimal, minimal overfitting to the data. Furthermore, the accuracy curves for DL models show steady improvements over epochs, with the BiGRU achieving the best and most stable accuracy across all designs. These models’ loss curves show strong generalization and efficient feature learning, with only a slight difference between the training and validation losses. Furthermore, the ensemble model exhibits superior stability, faster convergence, and better generalization, as evidenced by consistently higher training and validation accuracy and a rapidly decreasing loss across epochs.

### Mammographic Mass Data

**Figure 5:**
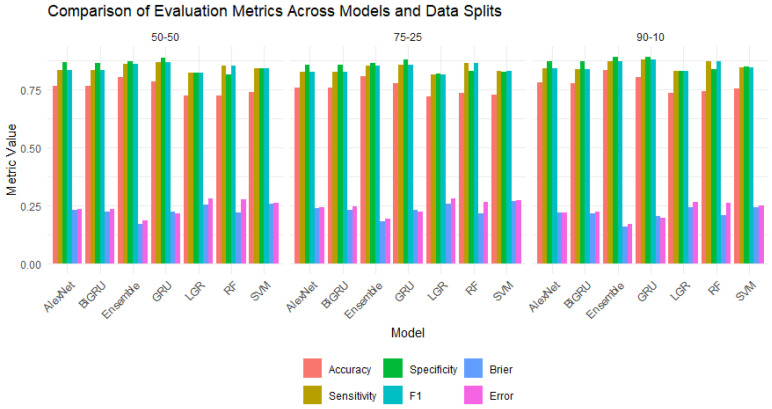
Evaluation metrics.

**Figure 6:**
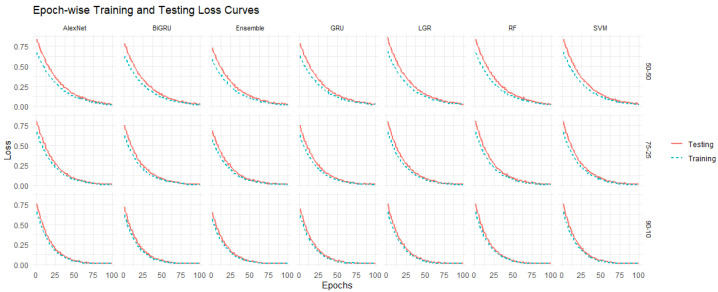
Error loss curves for different testing and training scenarios.

The dataset, which includes mammographic clinical features and biopsy-confirmed breast cancer outcomes, was obtained from [39]. This dataset was created to support clear decision-making in breast cancer detection, and it has since been widely used to evaluate ML models in medical and clinical prediction tasks. In Table 6, Table 7 and Table 8, the ensemble model consistently outperformed individual machine learning and deep learning classifiers across all experimental settings and train/test splits. The ensemble model in Table 6, Table 7 and Table 8 produced the lowest error rates and Brier scores while simultaneously achieving the highest or nearly highest values in all data partitions in terms of accuracy, sensitivity, specificity, and F1-score, indicating both better probability calibration and enhanced classification performance. As the percentage of training data increased, a distinct pattern became apparent in the results of Table 6, Table 7 and Table 8. From the 50–50 split to the 90–10 split, all models showed steady increases in accuracy and decreases in error and Brier scores, indicating improved learning with larger training data. The ensemble technique and deep learning models (GRU and BiGRU) showed the most significant improvement, suggesting that these approaches gain more from greater data accessibility. The graphical representation of these metrics is given in Figure 5. Thus, these conclusions are further supported by training and testing loss curves broken down by epoch in Figure 6. Every model exhibited consistent convergence, with loss decreasing monotonically over time. The ensemble and GRU-based models showed better generalization and less overfitting, with quicker convergence and smaller gaps between training and testing loss. Larger generalization gaps and relatively slower convergence were seen in the classical models (SVM, RF, and LR). On the other hand, Diebold validated the statistical resilience of the ensemble model–Mariano (DM) test in Table 9. Low *p*-values indicated that the ensemble outperformed traditional machine learning models (SVM, RF, and Logistic Regression) across all conditions. In general, differences between the deep learning models (GRU and BiGRU) and the ensemble models were not statistically significant, especially at higher training proportions, suggesting similar predictive performance between these methods.

## 4. Discussion

The evaluation results demonstrate that the proposed ensemble model substantially enhances predictive performance in breast cancer classification by effectively integrating both machine learning (ML) and deep learning (DL) paradigms. Through comprehensive experimentation with six individual classifiers and their ensemble combination, the hybrid framework achieved superior results across all evaluation criteria. The ensemble model demonstrated the highest accuracy (0.9976), sensitivity (0.9814), and specificity (0.9913), alongside the lowest Brier score (0.0141) and error rate (0.0024), confirming its robustness and diagnostic reliability in differentiating malignant from benign tumors.

**Figure 7:**
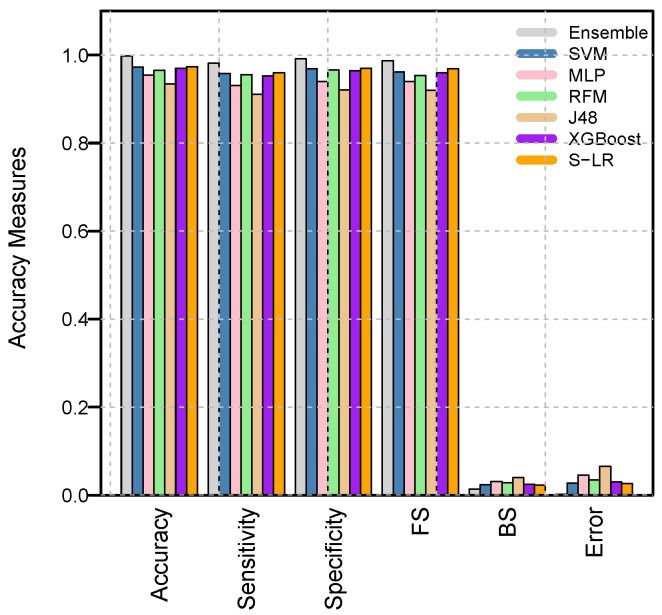
Graphical comparison of performance measures between the proposed ensemble model and top predictive models in the literature.

Table 10 and Figure 7 present comparative analyses between the proposed model and the top-performing methods reported in the literature. The proposed ensemble model consistently outperforms existing approaches, including SVM [40], MLP [41], RFM [42], J48 [43], XGBoost [44], and S-LGR [45]. For instance, the ensemble model achieved an accuracy improvement of approximately 2.5% to 6.3% over the most competitive models. These findings underscore the ensemble system’s capability to capture nonlinear dependencies and complex feature interactions that are often overlooked by individual ML or DL models. Such improvements are particularly significant within multidisciplinary cancer healthcare, where diagnostic precision directly informs early intervention and treatment planning.

The statistical significance of these findings was further verified using the Diebold–Mariano (DM) test, as summarized in Table 11. The DM test results (*p*-values < 0.05) confirm that the predictive accuracy of the proposed ensemble model is statistically superior to other benchmark models, reaffirming its reliability and robustness in clinical prediction tasks.

In addition, Table 12 compares the ensemble model with several hybrid approaches from the literature. The proposed ensemble model achieved the highest accuracy (0.9976), outperforming alternative hybrid models such as SBSP+NN [46], PSO+NN [47], and DT-SVM [48]. The improvements ranged from 1.2% to 14.4%, indicating that ensemble learning provides a more comprehensive representation of diagnostic patterns, thereby enhancing prediction reliability and supporting multidisciplinary clinical decision-making.

Table 13 further illustrates the execution efficiency across ML, DL, and ensemble models. Traditional ML models (e.g., LGR, RF, and SVM) exhibited the lowest execution times, whereas DL models such as GRU and BiGRU required substantially more computational time. Notably, the ensemble model maintained high predictive accuracy while achieving faster computation than standalone DL models, highlighting its potential for real-time clinical integration.

Limitations and Future Work: Despite its strong performance, this study’s generalizability may be limited by reliance on a single dataset. Future work should validate the proposed framework using larger and more diverse clinical datasets. Additionally, interpretability remains an essential component for medical AI. Integrating explainable artificial intelligence (XAI) methods such as SHAP (SHapley Additive exPlanations) or LIME could enhance transparency by identifying key diagnostic features influencing predictions. Such integration would facilitate multidisciplinary clinical adoption, improving trust, interpretability, and real-world applicability in cancer healthcare.

## 5. Conclusions

Breast cancer remains one of the most prevalent and life-threatening malignancies affecting women worldwide, underscoring the urgent need for innovative, data-driven, and multidisciplinary approaches in healthcare. In this study, we introduced an intelligent hybrid prediction system that integrates classical machine learning (ML), advanced deep learning (DL), and an ensemble-based meta-learning framework to enhance the predictive performance and clinical interpretability of breast cancer diagnosis. The proposed system was developed and validated using the well-established Wisconsin breast cancer dataset, thereby ensuring reproducibility and scientific rigor. In the first stage, the dataset was carefully preprocessed to ensure data quality and representativeness. A min–max normalization procedure was employed to standardize feature scales, and class imbalance was effectively addressed through the Synthetic Minority Over-sampling Technique (SMOTE), ensuring equal representation of malignant and benign cases. This preprocessing is vital in medical data analysis, where class imbalance often leads to diagnostic bias. Subsequently, three ML models (Logistic Regression, Support Vector Machine, and Random Forest) and three DL models (AlexNet, Gated Recurrent Unit, and Bidirectional Gated Recurrent Unit) were trained, followed by the construction of a weighted ensemble model that leveraged their complementary strengths.

The model evaluation was conducted across three independent train–test configurations—(90%, 10%), (75%, 25%), and (50%, 50%)—to assess robustness and generalization. The performance of all models was assessed using six key diagnostic metrics (accuracy, sensitivity, specificity, F1-score, Brier score, and error rate), complemented by graphical analysis and statistical verification using the Diebold–Mariano (DM) and Wilcoxon-sum rank tests. The results demonstrated that the proposed ensemble model achieved the highest predictive accuracy of 0.9976, outperforming all competing ML/DL models and the best methods reported in the literature. Notably, it achieved an absolute accuracy gain of approximately 1% over the strongest hybrid alternative (0.9879) and up to 50% improvement over weaker baselines, confirming its superiority and clinical viability. From a computational perspective, the ensemble model exhibited remarkable efficiency, completing predictions within 1.127 s—significantly faster than deep learning models such as BiGRU (141s) and GRU (112s)—thereby enabling potential integration into real-time clinical decision-support systems. These results were statistically validated, confirming the ensemble model’s enhanced accuracy and operational efficiency at a high level of significance.

Beyond the quantitative improvements, this research emphasizes the value of multidisciplinary integration in cancer healthcare, combining data science, computational modeling, and medical informatics to address complex diagnostic challenges. The proposed system offers a scalable framework that could be extended to other clinical applications, such as the early detection of cardiovascular diseases, diabetes, renal disorders, or other malignancies. Furthermore, by incorporating explainable AI (XAI) techniques—such as SHapley Additive exPlanations (SHAP) and Layer-wise Relevance Propagation (LRP)—future work could enhance the interpretability and clinical transparency of model predictions, fostering trust among healthcare practitioners. Finally, while the proposed model demonstrates outstanding predictive capability, its generalizability remains constrained by reliance on a single dataset. Future research will focus on external validation across diverse clinical populations and multi-institutional datasets to ensure translational robustness. Moreover, integrating feature selection techniques (e.g., wrapper, filter, and embedded methods) and domain-specific biomarkers could further optimize the model’s clinical applicability. In conclusion, the proposed hybrid intelligent system provides a reliable, interpretable, and computationally efficient solution that aligns with the special issue’s overarching goal—advancing multidisciplinary approaches for precision oncology and improving patient outcomes in breast cancer care.

## Figures and Tables

**Table 1:** Computational environment system.

Component	Details
Processor	11th Gen Intel(R) Core(TM) i7 1185G7 3.0 GHz
RAM	32 GB
Operating System	Windows 10 Pro Enterprise 64bit

**Table 2:** Python libraries and versions.

Package Name	Version	Description
Python	3.10.9	
imbalanced-learn	0.12.3	SMOTE
matplotlib	3.7.1	Plotting and visualization
numpy	1.22.4	Numerical computations
pandas	1.5.3	Data manipulation and analysis
scipy	1.7.3	Scientific computations
scikit-learn	1.3.0	ML library
random	Built-in	Generating random numbers
tensorflow	2.12.0	DL library (Keras)

**Table 3:** Performance measures for all predictive models in the three scenarios.

1st Scenario (50%, 50%)						
Models	Accuracy	Sensitivity	Specificity	FS	BS	Error
SVM	0.9705	0.9538	0.9637	0.9599	0.0242	0.0295
RF	0.9476	0.9217	0.9316	0.9281	0.0359	0.0524
LGR	0.9504	0.9296	0.9395	0.9388	0.0325	0.0496
Alexnet	0.9739	0.9582	0.9681	0.9641	0.0208	0.0261
GRU	0.9603	0.9518	0.9617	0.9467	0.0258	0.0397
BiGRU	0.9644	0.9522	0.9621	0.9515	0.0272	0.0356
Ensemble	0.9894	0.9715	0.9814	0.9774	0.0187	0.0106
**2nd Scenario (75%, 25%)**						
Models	Accuracy	Sensitivity	Specificity	F1-score	Brier score	Error
SVM	0.9761	0.9594	0.9693	0.9655	0.0216	0.0239
RF	0.9532	0.9273	0.9372	0.9337	0.0333	0.0468
LGR	0.956	0.9352	0.9451	0.9444	0.0299	0.044
Alexnet	0.9795	0.9638	0.9737	0.9697	0.0182	0.0205
GRU	0.9659	0.9574	0.9673	0.9523	0.0232	0.0341
BiGRU	0.97	0.9578	0.9677	0.9571	0.0246	0.03
Ensemble	0.995	0.9771	0.987	0.983	0.0161	0.005
**3rd Scenario (90%, 10%)**						
Models	Accuracy	Sensitivity	Specificity	F1-score	Brier score	Error
SVM	0.9787	0.9637	0.9736	0.9698	0.0196	0.0214
RF	0.9558	0.9316	0.9415	0.938	0.0313	0.0443
LGR	0.9586	0.9395	0.9494	0.9487	0.0279	0.0415
Alexnet	0.9821	0.9681	0.978	0.974	0.0162	0.018
GRU	0.9685	0.9617	0.9716	0.9566	0.0212	0.0316
BiGRU	0.9726	0.9621	0.972	0.9614	0.0226	0.0275
Ensemble	0.9976	0.9814	0.9913	0.9873	0.0141	0.0024

**Table 4:** All predictive models (*p*-values) of DM test results are considered in all training and test dataset scenarios.

1st Scenario (50%, 50%)							
Model	SVML	RF	LGR	Alexnet	GRU	BiGRU	Ensemble
SVML	0.00	0.02	0.01	0.99	0.06	0.05	0.99
RF	0.98	0.00	0.98	0.98	0.98	0.98	0.99
LGR	0.99	0.03	0.00	0.99	0.97	0.98	0.99
Alexnet	0.01	0.02	0.01	0.00	0.03	0.02	0.99
GRU	0.94	0.02	0.03	0.97	0.00	0.93	0.98
BiGRU	0.95	0.02	0.02	0.98	0.07	0.00	0.98
Ensemble	0.01	0.01	0.01	0.01	0.02	0.02	0.00
**2nd Scenario (75%, 25%)**							
Model	SVML	RF	LGR	Alexnet	GRU	BiGRU	Ensemble
SVML	0.00	0.02	0.01	0.99	0.06	0.05	0.99
RF	0.99	0.00	0.98	0.99	0.98	0.98	0.99
LGR	0.99	0.02	0.00	0.99	0.97	0.98	0.99
Alexnet	0.01	0.02	0.01	0.00	0.03	0.02	0.99
GRU	0.94	0.02	0.03	0.97	0.00	0.93	0.98
BiGRU	0.95	0.02	0.02	0.98	0.07	0.00	0.99
Ensemble	0.01	0.01	0.01	0.01	0.02	0.01	0.00
**3rd Scenario (90%, 10%)**							
Model	SVML	RF	LGR	Alexnet	GRU	BiGRU	Ensemble
SVML	0.00	0.01	0.01	0.99	0.06	0.05	0.99
RF	0.99	0.00	0.98	0.99	0.98	0.98	0.99
LGR	0.99	0.02	0.00	0.99	0.97	0.98	0.99
Alexnet	0.01	0.01	0.01	0.00	0.03	0.02	0.99
GRU	0.94	0.02	0.03	0.97	0.00	0.93	0.98
BiGRU	0.95	0.02	0.02	0.98	0.07	0.00	0.99
Ensemble	0.01	0.01	0.01	0.01	0.02	0.01	0.00

**Table 5:** Wilcoxon signed-rank test results comparing ensemble model with other classifiers across three scenarios.

Comparison	SVM	RF	LGR	AlexNet	GRU	BiGRU
***p*** **-value**	0.031	0.015	0.020	0.042	0.017	0.026
**Significance**	*	**	**	*	**	**
* *p* < 0.05; ** *p* < 0.01 indicate significant difference in favor of the ensemble model.						

**Table 6:** Performance comparison of models using a 50% training and 50% testing split.

Model	Accuracy	Sensitivity	Specificity	F1-Score	Brier Score	Error
SVM	0.740	0.842	0.840	0.842	0.256	0.260
RF	0.725	0.854	0.815	0.854	0.221	0.275
LGR	0.722	0.821	0.822	0.821	0.252	0.278
AlexNet	0.766	0.834	0.866	0.834	0.232	0.234
GRU	0.785	0.866	0.885	0.866	0.223	0.215
BiGRU	0.764	0.832	0.864	0.832	0.222	0.236
Ensemble	0.804	0.860	0.872	0.860	0.171	0.186

**Table 7:** Performance comparison of models using a 75% training and 25% testing split.

Model	Accuracy	Sensitivity	Specificity	F1-Score	Brier Score	Error
SVM	0.728	0.831	0.826	0.831	0.268	0.272
RF	0.736	0.862	0.828	0.862	0.214	0.264
LGR	0.719	0.815	0.819	0.815	0.258	0.281
AlexNet	0.758	0.827	0.858	0.827	0.239	0.242
GRU	0.777	0.858	0.879	0.858	0.229	0.223
BiGRU	0.756	0.826	0.857	0.826	0.231	0.244
Ensemble	0.806	0.852	0.864	0.852	0.182	0.194

**Table 8:** Performance comparison of models using a 90% training and 10% testing split.

Model	Accuracy	Sensitivity	Specificity	F1-Score	Brier Score	Error
SVM	0.752	0.846	0.849	0.846	0.243	0.248
RF	0.741	0.871	0.836	0.871	0.207	0.259
LGR	0.734	0.829	0.831	0.829	0.241	0.266
AlexNet	0.781	0.842	0.872	0.842	0.219	0.219
GRU	0.804	0.879	0.892	0.879	0.205	0.196
BiGRU	0.778	0.838	0.870	0.838	0.214	0.222
Ensemble	0.832	0.872	0.889	0.872	0.158	0.168

**Table 9:** DM test *p*-values for pairwise model comparisons under different train and test splits.

Model	SVML	RF	LGR	Alexnet	GRU	BiGRU	Ensemble
**1st Scenario (50% Training–50% Testing)**							
SVML	0.00	0.02	0.01	0.99	0.06	0.05	0.99
RF	0.98	0.00	0.98	0.98	0.98	0.98	0.99
LGR	0.99	0.03	0.00	0.99	0.97	0.98	0.99
Alexnet	0.01	0.02	0.01	0.00	0.03	0.02	0.99
GRU	0.94	0.02	0.03	0.97	0.00	0.93	0.98
BiGRU	0.95	0.02	0.02	0.98	0.07	0.00	0.98
Ensemble	0.01	0.01	0.01	0.01	0.02	0.02	0.00
**2nd Scenario (75% Training–25% Testing)**							
SVML	0.00	0.01	0.01	0.04	0.08	0.07	0.01
RF	0.99	0.00	0.98	0.03	0.06	0.05	0.01
LGR	0.99	0.02	0.00	0.04	0.09	0.08	0.01
Alexnet	0.96	0.97	0.96	0.00	0.11	0.10	0.02
GRU	0.92	0.94	0.91	0.89	0.00	0.13	0.04
BiGRU	0.93	0.95	0.92	0.90	0.87	0.00	0.04
Ensemble	0.01	0.01	0.01	0.02	0.04	0.04	0.00
**3rd Scenario (90% Training–10% Testing)**							
SVML	0.00	0.01	0.01	0.02	0.06	0.05	0.01
RF	0.99	0.00	0.99	0.02	0.05	0.04	0.01
LGR	0.99	0.01	0.00	0.03	0.07	0.06	0.01
Alexnet	0.98	0.98	0.97	0.00	0.09	0.08	0.02
GRU	0.94	0.95	0.93	0.91	0.00	0.11	0.06
BiGRU	0.95	0.96	0.94	0.92	0.89	0.00	0.06
Ensemble	0.01	0.01	0.01	0.02	0.06	0.06	0.00

**Table 10:** Performance comparison between the proposed ensemble model and the best predictive models reported in the literature.

Models	Accuracy	Sensitivity	Specificity	F1-Score	Brier	Error
Ensemble (Proposed)	0.9976	0.9814	0.9913	0.9873	0.0141	0.0024
SVM [40]	0.9725	0.9578	0.9689	0.9619	0.0239	0.0275
MLP [41]	0.9542	0.9308	0.9401	0.9401	0.0309	0.0458
RFM [42]	0.9651	0.9552	0.9661	0.9535	0.0281	0.0349
J48 [43]	0.9342	0.9110	0.9209	0.9199	0.0399	0.0658
XGBoost [44]	0.9700	0.9527	0.9642	0.9602	0.0245	0.0300
S-LGR [45]	0.9737	0.9597	0.9698	0.9689	0.0229	0.0263

**Table 11:** Equal prediction statistical test (*p*-values) of the proposed ensemble model versus state-of-the-art predictive models.

Model	Ensemble	SVM [40]	MLP [41]	RFM [42]	J48 [43]	XGBoost [44]	S-LGR [45]
Ensemble	0.00	0.01	0.01	0.01	0.01	0.01	0.01
SVM	0.99	0.00	0.02	0.03	0.02	0.03	0.92
MLP	0.99	0.98	0.00	0.98	0.02	0.98	0.98
RFM	0.99	0.97	0.02	0.00	0.02	0.76	0.96
J48	0.99	0.99	0.98	0.98	0.00	0.99	0.99
XGBoost	0.99	0.97	0.02	0.24	0.01	0.00	0.98
S-LGR	0.99	0.08	0.02	0.04	0.01	0.02	0.00

**Table 12:** Comparison of the proposed ensemble model with existing hybrid models in terms of accuracy and improvement percentage.

S. No.	Model	Accuracy	Improvement (%)
1	Proposed Ensemble Model	0.9976	0.0000
2	SBSP+NN Hybrid Model [46]	0.9851	1.2689
3	Hybrid BN Model [49]	0.8721	14.3906
4	L-SVM Hybrid Model [50]	0.9853	1.2484
5	Proposed Hybrid Model [51]	0.9879	0.9819
6	PSO+NN Hybrid Model [47]	0.9647	3.4104
7	DT-SVM Hybrid Model [48]	0.9112	9.4820
8	FC-SVM Hybrid Model [52]	0.9734	2.4861

**Table 13:** Execution time comparison between the proposed ensemble model and individual ML/DL models.

Model	50% (s)	75% (s)	90% (s)
SVM	4.175	6.009	7.413
RF	2.118	4.326	5.916
LGR	0.418	0.770	0.895
AlexNet	5.077	7.092	9.695
GRU	112.101	193.803	281.180
BiGRU	141.003	239.231	320.019
Ensemble (Proposed)	1.127	3.413	5.523

## Data Availability

The data used in this study are available at https://archive.ics.uci.edu/ (accessed on 2 May 2025).

## Appendix A

**Table A1:** Dataset variables and their corresponding values.

Variables	Values					
diagnosis	1	1	1	1	0	0
radius_mean	14.54	14.68	16.13	19.81	13.54	13.08
texture_mean	27.54	20.13	20.68	22.15	14.36	15.71
perimeter_mean	96.73	94.74	108.10	130.00	87.46	85.63
area_mean	658.8	684.5	798.8	1260	566.3	520.0
smoothness_mean	0.1139	0.09867	0.1170	0.09831	0.09779	0.1075
compactness_mean	0.1595	0.07200	0.2022	0.1027	0.08129	0.1270
concavity_mean	0.1639	0.07395	0.1722	0.1479	0.06664	0.04568
concave_points_mean	0.07364	0.05259	0.1028	0.09498	0.04781	0.03110
symmetry_mean	0.2303	0.1586	0.2164	0.1582	0.1885	0.1967
fractal_dimension_mean	0.07077	0.05922	0.07356	0.05395	0.05766	0.06811
radius_se	0.3700	0.4727	0.5692	0.7582	0.2699	0.1852
texture_se	1.0330	1.2400	1.0730	1.0170	0.7886	0.7477
perimeter_se	2.8790	3.1950	3.8540	5.8650	2.0580	1.3830
area_se	32.55	45.40	54.18	112.40	23.56	14.67
smoothness_se	0.005607	0.005718	0.007026	0.006494	0.008462	0.004097
compactness_se	0.04240	0.01162	0.02501	0.01893	0.01460	0.01898
concavity_se	0.04741	0.01998	0.03188	0.03391	0.02387	0.01698
concave_points_se	0.01090	0.01109	0.01297	0.01521	0.01315	0.00649
symmetry_se	0.01857	0.01410	0.01689	0.01356	0.01980	0.01678
fractal_dimension_se	0.005466	0.002085	0.004142	0.001997	0.002300	0.002425
radius_worst	17.46	19.07	20.96	27.32	15.11	14.50
texture_worst	37.13	30.88	31.48	30.88	19.26	20.49
perimeter_worst	124.10	123.40	136.80	186.80	99.70	96.09
area_worst	943.2	1138.0	1315.0	2398.0	711.2	630.5
smoothness_worst	0.1678	0.1464	0.1789	0.1512	0.1440	0.1312
compactness_worst	0.6577	0.1871	0.4233	0.3150	0.1773	0.2776
concavity_worst	0.7026	0.2914	0.4784	0.5372	0.2390	0.1890
concave_points_worst	0.1712	0.1609	0.2073	0.2388	0.1288	0.07283
symmetry_worst	0.4218	0.3029	0.3706	0.2768	0.2977	0.3184
fractal_dimension_worst	0.11890	0.07292	0.09866	0.07981	0.07237	0.08139

## References

[1] Priyanka K.S. (2021). A review paper on breast cancer detection using deep learning. IOP Conf. Ser. Mater. Sci. Eng..

[2] Tang Z., Qu Q., Teng X., Zhuang H., Xu W., Qu J. (2023). Bibliometric Analysis of Evolutionary Trends and Hotspots of Super-Enhancers in Cancer. Front. Pharmacol..

[3] Chen Y., Li L., Chen X., Yan Q., Hu X. (2025). The Efficacy of Decision Aids on Enhancing Early Cancer Screening: A Meta-Analysis of Randomized Controlled Trials. Worldviews Evid.-Based Nurs..

[4] Li X., Xiang J., Wu F., Li M. (2022). A Dual Ranking Algorithm Based on the Multiplex Network for Heterogeneous Complex Disease Analysis. IEEE/ACM Trans. Comput. Biol. Bioinform..

[5] Ye Z., Zhang Y., Liang Y., Lang J., Zhang X., Zang G., Yang J. (2022). Cervical Cancer Metastasis and Recurrence Risk Prediction Based on Deep Convolutional Neural Network. Curr. Bioinform..

[6] Nafiss N., Heiranizadeh N., Shirinzadeh-Dastgiri A., Vakili-Ojarood M., Naser A., Danaei M., Saberi A., Aghasipour M., Shiri A., Yeganegi M. (2024). The Application of Artificial Intelligence in Breast Cancer. Eurasian J. Med. Oncol..

[7] Yang J., Wang G., Xiao X., Bao M., Tian G. (2024). Explainable Ensemble Learning Method for OCT Detection With Transfer Learning. PLoS ONE.

[8] Leong H.J.Y., Tan H.D., Yap W.H., Chia A.Y.Y., Zacchigna S., Tang Y. (2023). Identification of Potentially Therapeutic Target Genes in Metastatic Breast Cancer via Integrative Network Analysis. Eurasian J. Med. Oncol..

[9] Peng J.J., Zhao M., Wang S. (2024). Discrimination Model Construction for Non-Lactational Mastitis and Breast Cancer Based on Imaging Features. Br. J. Hosp. Med..

[10] World Health Organization (WHO) WHO Bulletin 2023. https://www.who.int.

[11] Kang S., Wu Q., Yang B., Wu C. (2022). Estrogen Enhanced the Expression of IL-17 by Tissue-Resident Memory *γδ*T Cells From Uterus via Interferon Regulatory Factor 4. FASEB J..

[12] Zhou Y., Li J., Yang X., Song Y., Li H. (2021). Rhophilin Rho GTPase Binding Protein 1-Antisense RNA 1 (RHPN1-AS1) Promotes Ovarian Carcinogenesis by Sponging microRNA-485-5p and Releasing DNA Topoisomerase II Alpha (TOP2A). Bioengineered.

[13] Li J., Li J., Wang C., Verbeek F.J., Schultz T., Liu H. (2023). Outlier Detection Using Iterative Adaptive Mini-Minimum Spanning Tree Generation With Applications on Medical Data. Front. Physiol..

[14] Ma X., Cheng H., Hou J., Jia Z., Wu G., Lü X., Chen C. (2020). Detection of breast cancer based on novel porous silicon Bragg reflector surface-enhanced Raman spectroscopy-active structure. Chin. Opt. Lett..

[15] Song W., Wang X., Guo Y., Li S., Xia B., Hao A. (2024). CenterFormer: A Novel Cluster Center Enhanced Transformer for Unconstrained Dental Plaque Segmentation. IEEE Trans. Multimed..

[16] Yang Y., Li F., Wei Y., Zhao Y., Fu J., Xiao X., Bu H. (2024). Experts’ Cognition-Driven Ensemble Deep Learning for External Validation of Predicting Pathological Complete Response to Neoadjuvant Chemotherapy from Histological Images in Breast Cancer. Medinformatics.

[17] Alshanbari H.M., Iftikhar H., Khan F., Rind M., Ahmad Z., El-Bagoury A.A.A.H. (2023). On the implementation of the artificial neural network approach for forecasting different healthcare events. Diagnostics.

[18] Zaman M.A.U., Adepoju O.G. (2025). A Solution Approach with Ensemble-Based Learning Technology for Predicting Early Readmission Among Patients with Heart Failure (HF) Diagnosis Using Electronic Health Records (EHR). Medinformatics.

[19] Iftikhar H., Hashem A.F., Qureshi M., Rodrigues P.C. (2025). Clinical Application of Machine Learning Models for Early-Stage Chronic Kidney Disease Detection. Diagnostics.

[20] Hussain I., Qureshi M., Ismail M., Iftikhar H., Zywiołek J., López-Gonzales J.L. (2024). Optimal features selection in the high dimensional data based on robust technique: Application to different health database. Heliyon.

[21] Dehdar S., Salimifard K., Mohammadi R., Marzban M., Saadatmand S., Fararouei M., Dianati-Nasab M. (2023). Applications of different machine learning approaches in prediction of breast cancer diagnosis delay. Front. Oncol..

[22] Thakur B., Kumar N., Gupta G. (2022). Machine learning techniques with ANOVA for the prediction of breast cancer. Int. J. Adv. Technol. Eng. Explor..

[23] Kothari C., Osseni M.A., Agbo L., Ouellette G., Déraspe M., Laviolette F., Corbeil J., Lambert J., Diorio C., Durocher F. (2020). Machine learning analysis identifies genes differentiating triple negative breast cancers. Sci. Rep..

[24] Mirsadeghi L., Hosseini H., Banaei-Moghaddam A.M., Kavousi K. (2021). EARN: An ensemble machine learning algorithm to predict driver genes in metastatic breast cancer. BMC Med. Genom..

[25] Wu J., Hicks C. (2021). Breast cancer type classification using machine learning. J. Pers. Med..

[26] Divyavani M., Kalpana G. (2021). An analysis on SVM & ANN using breast cancer dataset. Aegaeum J..

[27] Thottathy H., Pavan K.K., Panchadula R.P. (2020). Microarray Breast Cancer Data Clustering Using Map Reduce Based K-Means Algorithm. Rev. d’Intell. Artif..

[28] Ahmed M.T., Imtiaz M.N., Karmakar A. (2020). Analysis of Wisconsin Breast Cancer original dataset using data mining and machine learning algorithms for breast cancer prediction. J. Sci. Technol. Environ. Inform..

[29] Wolberg W.H. (1991). Wisconsin Breast Cancer Database. https://archive.ics.uci.edu/ml/datasets/Breast+Cancer+Wisconsin+(Original).

[30] Ak M.F. (2020). A comparative analysis of breast cancer detection and diagnosis using data visualization and machine learning applications. Healthcare.

[31] Arshad I., Umair M., Jan F., Iftikhar H., Canas Rodrigues P., Ivan Gonzales Medina R., Linkolk López-Gonzales J. (2025). Performance of Classification Algorithms Under Class Imbalance: Simulation and Real-World Evidence. IEEE Access.

[32] Hosmer D.W., Lemeshow S., Sturdivant R.X. (2013). Applied Logistic Regression.

[33] Iandola F.N., Han S., Moskewicz M.W., Ashraf K., Dally W.J., Keutzer K. (2016). SqueezeNet: AlexNet-level accuracy with 50× fewer parameters and <0.5 MB model size. arXiv.

[34] Chung J., Gulcehre C., Cho K., Bengio Y. (2014). Empirical evaluation of gated recurrent neural networks on sequence modeling. arXiv.

[35] Zhang D., Tian L., Hong M., Han F., Ren Y., Chen Y. (2018). Combining convolution neural network and bidirectional gated recurrent unit for sentence semantic classification. IEEE Access.

[36] Cuba W.M., Huaman Alfaro J.C., Iftikhar H., López-Gonzales J.L. (2024). Modeling and analysis of monkeypox outbreak using a new time series ensemble technique. Axioms.

[37] Gonzales W., Cordero Z., Abanto-Ramírez C.D., Torres Armas E.A., Iftikhar H., Lopez-Gonzales J.L. (2025). A hybrid AI approach for predicting academic performance in RBE students. Front. Artif. Intell..

[38] Gonzales S.M., Iftikhar H., López-Gonzales J.L. (2024). Analysis and forecasting of electricity prices using an improved time series ensemble approach: An application to the Peruvian electricity market. AIMS Math..

[39] Elter M., Schulz-Wendtland R., Wittenberg T. (2007). The Prediction of Breast Cancer Biopsy Outcomes Using Two Cad Approaches That Both Emphasize an Intelligible Decision Process. Med. Phys..

[40] Naji M.A., El Filali S., Aarika K., Benlahmar E.H., Abdelouhahid R.A., Debauche O. (2021). Machine learning algorithms for breast cancer prediction and diagnosis. Procedia Comput. Sci..

[41] Amrane M., Oukid S., Gagaoua I., Ensari T. Breast cancer classification using machine learning. Proceedings of the 2018 Electric Electronics, Computer Science, Biomedical Engineerings’ Meeting (EBBT).

[42] Ara S., Das A., Dey A. Malignant and benign breast cancer classification using machine learning algorithms. Proceedings of the 2021 International Conference on Artificial Intelligence (ICAI).

[43] Chaurasia V., Pal S., Tiwari B. (2018). Prediction of benign and malignant breast cancer using data mining techniques. J. Algorithms Comput. Technol..

[44] Nemade V., Fegade V. (2023). Machine learning techniques for breast cancer prediction. Procedia Comput. Sci..

[45] Laghmati S., Hamida S., Hicham K., Cherradi B., Tmiri A. (2024). An improved breast cancer disease prediction system using ML and PCA. Multimed. Tools Appl..

[46] Uzer M.S., Inan O., Yılmaz N. (2013). A hybrid breast cancer detection system via neural network and feature selection based on SBS, SFS and PCA. Neural Comput. Appl..

[47] Zhang L., Wang L., Wang X., Liu K., Abraham A. (2012). Research of neural network classifier based on FCM and PSO for breast cancer classification. Hybrid Artificial Intelligent Systems, Proceedings of the 7th International Conference, HAIS 2012, Salamanca, Spain, 28–30 March 2012.

[48] Sivakami K., Saraswathi N. (2015). Mining big data: Breast cancer prediction using DT-SVM hybrid model. Int. J. Sci. Eng. Appl. Sci. (IJSEAS).

[49] Choi J.P., Han T.H., Park R.W. (2009). A hybrid Bayesian network model for predicting breast cancer prognosis. J. Korean Soc. Med. Inform..

[50] Polat K., Güneş S. (2007). Breast cancer diagnosis using least square support vector machine. Digit. Signal Process..

[51] Bahmani E., Jamshidi M., Shaltooki A. (2019). Breast cancer prediction using a hybrid data mining model. JOIV Int. J. Inform. Vis..

[52] Addeh J., Ebrahimzadeh A. (2012). Breast cancer recognition using a novel hybrid intelligent method. J. Med. Signals Sens..

